# Deciphering the Molecular Adapting Mechanism of Lactic Acid-Tolerant *Saccharomyces cerevisiae* Through Genomic and Transcriptomic Analysis

**DOI:** 10.3390/foods14122027

**Published:** 2025-06-08

**Authors:** Haowei Fan, Yin Wan, Wenqin Cai, Feng Li, Jiahui Fan, Juan Du, Mingjing Yi, Jiayi Yuan, Guiming Fu

**Affiliations:** 1 State Key Laboratory of Food Science and Resources, College of food Science and Technology, Nanchang University, Nanchang 330047, China; fanhaowei@email.ncu.edu.cn (H.F.); yinwan@ncu.edu.cn (Y.W.); 18166043836@163.com (W.C.); 417900230036@email.ncu.edu.cn (F.L.); fjh18638620253@163.com (J.F.); 13639189937@163.com (J.D.); ymj652681652@gmail.com (M.Y.); 407900230119@email.ncu.edu.cn (J.Y.); 2 International Institute of Food Innovation, Nanchang University, Nanchang 330047, China

**Keywords:** *Saccharomyces cerevisiae*, lactic acid tolerance, genomics, transcriptomics

## Abstract

During the solid-state brewing process of traditional Chinese Baijiu, lactic acid is the most abundant organic acid, which inhibits the growth and metabolism of *Saccharomyces cerevisiae*. To reveal the lactic acid tolerance mechanism of *S. cerevisiae*, the growth, metabolic performance, and antioxidant enzyme activity of *S. cerevisiae* NCUF309.5-44 and *S. cerevisiae* NCUF309.5 were measured under 4% (*v*/*v*) lactic acid stress. Additionally, whole-genome re-sequencing and transcriptomic analyses were performed to identify genetic variations and differentially expressed genes between the two strains under lactic acid stress. The results showed that, compared to the original strain, *S. cerevisiae* NCUF309.5-44 could adapt to the lactic acid stress faster, with a superior utilization rate of reducing sugar and a 6.43-fold higher ethanol production at 16 h. The strain primarily activated the GSH/GPx system, resulting in a 37.29% lower intracellular ROS content. A total of 1087 SNPs and 698 InDels were found between the strains, with 384 genes significantly upregulated and 254 genes downregulated in the *S. cerevisiae* NCUF309.5-44 under lactic acid stress. *S. cerevisiae* NCUF309.5-44 responded to lactic acid stress by activating the pheromone response pathway and the cell wall integrity pathway. Meanwhile, the capacity of strains to maintain the cell membrane and proton extrusion was strengthened. Additionally, its glycolysis/gluconeogenesis metabolism was also enhanced. All these mechanisms collectively contributed to improving the lactic acid tolerance of *S. cerevisiae* NCUF309.5-44. These findings not only enhanced our understanding of lactic acid tolerance mechanisms of *S. cerevisiae* NCUF309.5-44 but also paved the way for the application of this strain in optimizing *Baijiu* production.

## 1. Introduction

During traditional Chinese *Baijiu* production, the fermentation and distillation processes occur in the absence of significant free liquid, which is called solid-state *Baijiu* brewing. *Jiupei* is the fermented mixture obtained by mixing steamed grains as a fermentation starter, and its environmental factors are constantly in a state of dynamic change in the brewing process [1]. As the dominant microorganisms in the fermentation process of *Jiupei*, *Saccharomyces cerevisiae* can be affected by multiple environmental stress factors, including organic acids, ethanol, temperature fluctuations, and osmotic pressure, leading to issues like reduced fermentation rates and decreased degradation of reducing sugars, ultimately impacting fermentation efficiency and severely downgrade the quality of the final product [2]. Lactic acid bacteria are among the most abundant fermentative microorganisms in the pit, continuously producing and accumulating lactic acid. As a result, lactic acid becomes the most prevalent organic acid in the middle and late stage (aroma-producing stage) of Chinese solid-state *Baijiu* brewing, with its content reaching up to 3.62% ± 0.62% in *Jiupei* [3], which is also the reason why we selected a 4% lactic acid concentration for the experiments. While lactic acid serves as a precursor for flavor compound synthesis, excessive concentrations severely inhibit the normal growth and metabolism of yeast. Therefore, improving the lactic acid tolerance of *S. cerevisiae* and elucidating its tolerance mechanisms have become issues that need to be addressed in the traditional Chinese solid-state *Baijiu* brewing process. In our previous study, the *S. cerevisiae* NCUF309.5-44 that could tolerate 4% lactic acid stress through Atmospheric and Room Temperature Plasma (ARTP) mutagenesis combined with Microbial Microdroplet Culture (MMC) treatment was obtained [4]. However, the mechanism of its lactic acid tolerance remains unclear.

Currently, the literature on the potential mechanisms of yeast tolerance to acid stress mainly focuses on biomass conversion and the industrial production of organic acids. Zeng et al. [5] reported that yeast could enhance its formic acid tolerance and achieve self-protection during lignocellulosic conversion by slowing the synthesis of certain amino acids and nucleotides and reducing energy consumption. On the other hand, Ribeiro et al. [6] found that yeast could alter the structure of the cell wall to increase its hardness, thereby improving its tolerance to acetic acid stress. In addition, the simultaneous activation of biotransformation, stress response, and transmembrane transport, along with the transcription factors Haa1p, Hap4p, Yox1p, and Mag1p to enhance formic and acetic acid tolerance in yeast cells, was also observed [7]. Additionally, Hakkaart et al. [8] also revealed the detrimental effects of low pH on yeast strains used in industrial applications, including increased energy maintenance requirements and a rise in mortality rates. However, these studies primarily focused on the tolerance mechanisms of engineered and wild strains to acid stress in industrial production, and there is still a relative lack of research on gene mutant strains that could tolerate high concentrations of acid stress. More critically, this research direction will lay the foundation for future research on lactic acid tolerance in yeast strains and directly benefit the Chinese traditional *Baijiu* production industry.

In recent years, the rapid development of various omics technologies has provided important tools for studying the relationship between genes and phenotypes of microorganisms. However, single-omics technology can only provide data on nucleic acids, proteins, or metabolites, without offering insights into the complex metabolic networks of microorganisms [9]. To reveal the tolerance mechanisms of yeast, multi-omics approaches are typically employed for research. The combined analysis of genomics and transcriptomics has been proven to comprehensively reveal the tolerance mechanisms of microorganisms to 2-phenylethanol stress [10]. Fletcher et al. [11] also utilized a multi-omics strategy to confirm that there were significant differences in the adaptability of *S. cerevisiae* obtained through evolutionary engineering methods to various acids. Similarly, by combining multi-omics techniques with the analysis of metabolic and aromatic compounds, the key genes related to acid-tolerant *S. cerevisiae* used in the fermentation of greengage wine were identified, and the pathways regulating the formation of aromatic compounds were discovered [12].

Therefore, this study employed a combination of genomic and transcriptomic analyses to investigate the changes in genes and metabolic pathways between the lactic acid-tolerant *S. cerevisiae* NCUF309.5-44 and the original strain *S. cerevisiae* NCUF309.5. By delving into key acid tolerance genes and pathways, this study laid the groundwork for applying S. cerevisiae NCUF309.5-44 in traditional Chinese solid-state Baijiu brewing, which could enhance fermentation processes in *Jiupei* and result in higher quality and yield. Additionally, this research provided a conceptual foundation for developing lactic acid-tolerant genetically engineered yeast strains, facilitating the creation of robust strains for bioethanol production.

## 2. Materials and Methods

### 2.1. Materials and Culture

The strain *S. cerevisiae* NCUF309.5 employed in this research was isolated from *Te*-flavor *Baijiu Daqu* (from a *Te*-flavor *Baijiu* brewing enterprise in Zhangshu, China). Its growth was affected by the low concentration of 0.5% lactic acid and was significantly inhibited by the concentration of 4% lactic acid. The lactic acid-tolerant strain *S. cerevisiae* NCUF309.5-44 capable of tolerating lactic acid stress up to 4% was obtained from *S. cerevisiae* NCUF309.5 through ARTP (ARTP-IIS, Wuxi Tmaxtree Biotechnology Co., Ltd., Wuxi, China) mutagenesis combined with MMC (MMC-B2, Wuxi Tmaxtree Biotechnology Co., Ltd., Wuxi, China) treatment [4]. The original strain and lactic acid-tolerant strain were all maintained at the State Key Laboratory of Food Science and Resources of Nanchang University. The preserved yeast strains were activated and cultured in YPD medium (1% yeast extract, 2% peptone, and 2% glucose, purchased from Huankai Microbial Technology Co., Ltd., Guangzhou, China) to prepare the seed culture. The seed culture was then inoculated into a YPD medium supplemented with 4% lactic acid and incubated at 28 °C, 160 rpm, with an initial inoculum of 10^6^ CFU/mL. All the chemicals employed in this research were of analytical grade.

### 2.2. Measurement of Growth, Reducing Sugar Content, and Ethanol Content of S. cerevisiae Under Lactic Acid Stress

*S. cerevisiae* was inoculated at a concentration of 1 × 10^6^ cells into 100 mL YPD medium containing 4% lactic acid and cultivated at 28 °C, 160 rpm. Samples were taken every 4 h, and the growth of the cells was calculated using the hemocytometer method under an optical microscope. The cells were observed under a 40× objective lens, and the number of cells in the top-left, bottom-left, top-right, bottom-right, and central squares of the hemocytometer was counted. The number of cells was calculated using the following formula:

$$Number\;of\;cells\;\left(CFU/mL\right)=X\times 5\times 10^{6}\times dilution\;factor$$

where *X* is the total number of cells in 5 squares.

The 3, 5-dinitrosalicylic acid (DNS) colorimetric method was used to determine the content of reducing sugar in a culture medium according to Li et al. [13]. The determination of ethanol content was carried out using gas chromatography (GC) (8860, Agilent Technologies, Santa Clara, CA, USA), as described by Yang et al. [14].

### 2.3. Measurement of Reactive Oxygen Species (ROS) Content of S. cerevisiae Under Lactic Acid Stress

The ROS content was measured using a ROS Assay Kit (Shanghai Beyotime Biotechnology Co., Ltd., Shanghai, China). The treatment was carried out according to the method of Kang et al. [15]. An amount of 1 mL of the fermentation broth was collected every 8 h, centrifuged to collect the yeast cells, and then resuspended in a diluted DCFH-DA solution. The cells were kept at 37 °C for a 20 min incubation period. Subsequently, the yeast cells were subjected to a triple washing procedure with sterile water to remove the unincorporated DCFH-DA. Finally, fluorescence intensity was quantified using a microplate reader (Varioskan LUX, Thermo Fisher Scientific Inc., Waltham, MA, USA) setting the excitation at 488 nm and the detection at 525 nm. ROS content was represented by the fluorescence intensity.

### 2.4. Measurement of Antioxidant Enzyme Activity of S. cerevisiae Under Lactic Acid Stress

The method depicted by Chen et al. [16] was used to measure the antioxidant enzyme activity in *S. cerevisiae* 309.5-44 and *S. cerevisiae* 309.5 under the 4% lactic acid stress. Briefly, every 8 h, 10 mL of the culture medium was collected and centrifuged to harvest the yeast cells, which were then washed and resuspended three times in PBS. The cell suspension was placed on ice and subjected to ultrasonic disruption for 300 s (60 times, 5 s each, with a 5 s interval). Following centrifugation at 4 °C, 12,000× *g*, for a duration of 10 min, the upper fraction was collected for subsequent determination of antioxidant enzyme activity. The activities of superoxide dismutase (SOD), catalase (CAT), and glutathione peroxidase (GPx) were determined using antioxidant enzyme activity analysis kits (Nanjing Jiancheng Bioengineering Institute, Nanjing, China) adhering to the protocols provided by the manufacturer. The activities of the antioxidant enzymes were expressed as U/g.

### 2.5. Re-Sequencing of Whole Genome

#### 2.5.1. Genomic DNA Library Preparation and Sequencing

The method for the re-sequencing of the whole genome was based on the description by Tian et al., with some modifications [12]. An amount of 20 mL of the culture medium was collected and centrifuged at 12,000× *g* for 15 min at 4 °C when cultured to 16 h. The cell pellets were subsequently rinsed thrice using phosphate-buffered saline (PBS). Following the washes, the cells were promptly immersed in liquid nitrogen and preserved at a temperature of −80 °C for the re-sequencing of the whole genome and transcriptomic analyses. The CTAB method was employed to extract the DNA of *S. cerevisiae* NCUF309.5 and NCUF309.5-44. Only DNA samples of superior quality (OD260/280 = 1.8~2.0, OD260/230 ≥ 2.0) were selected for the assembly of the sequencing library. The sequencing library was generated using Truseq Nano DNA HT Sample Prep Kit (Illumina, San Diego, CA, USA) following the manufacturer’s recommendations, and index codes were added to each sample. After PCR products were purified (AMPure XP system, Beckman Coulter, Inc., Brea, CA, USA), libraries were analyzed for size distribution by Agilent 2100 Bioanalyzer and quantified by real-time PCR (3 nM). The paired-end DNA-seq sequencing library was subjected to sequencing on the Illumina NovaSeq platform by Shanghai Majorbio Bio-pharm Technology Co., Ltd., Shanghai, China. The whole-genome re-sequencing data have been submitted to the NCBI database (BioProject: PRJNA1224395).

#### 2.5.2. Variant Discovery

Raw reads that were of low quality were either trimmed or discarded using the Fastp software [17]. The clean reads obtained from the two strains were mapped to the designated genome reference of S. cerevisiae strain S288c (accession number: SAMD00065885) using BWA-MEME software [18]. Single-nucleotide polymorphisms (SNPs) and insertions/deletions (InDels) were classified according to their genomic locations [10].

### 2.6. Transcriptomic

#### 2.6.1. RNA Extraction

Total RNA was isolated from the tissue with TRIzol^®^ Reagent, following the protocol provided by the manufacturer. The RNA quality was assessed using the 5300 Bioanalyzer (Agilent Technologies, CA, USA), and the RNA concentration was measured with the ND-2000 Spectrophotometer (NanoDrop Technologies, Edison, NJ, USA). For the construction of the sequencing library, only RNA samples of high quality were used (OD_260/280_ = 1.8~2.2, OD_260/230_ ≥ 2.0, RQN ≥ 6.5).

#### 2.6.2. Library Preparation and Sequencing

Library preparation and sequencing were performed at Shanghai Majorbio Bio-pharm Biotechnology Co., Ltd. (Shanghai, China) according to the manufacturer’s instructions. The *S. cerevisiae* NCUF309.5 and NCUF309.5-44 RNA-seq transcriptome library was prepared following Illumina^®^ Stranded mRNA Prep, Ligation (San Diego, CA, USA) using 1 μg of total RNA. After quantified by Qubit 4.0, the sequencing library was carried out using NovaSeq Reagent Kit (Illumina, CA, USA). The original data have been uploaded to NCBI (BioProject: PRJNA1224709).

#### 2.6.3. Differential Expression Analysis and Functional Enrichment

To identify differentially expressed genes (DEGs) between two strains, the expression level of each transcript was quantified using the transcripts per million (TPM) method. DEGs with a *p*-adjusted value of ≤0.05 and a value of the logarithm of Fold Change with base 2 ((|log2Fold Change|) > 2) were identified as significantly different expressed genes. Gene Ontology (GO) functional enrichment and Kyoto Encyclopedia of Genes and Genomes (KEGG) pathway analysis were carried out by Goatools 0.6.5 and KOBAS 2.1.1, respectively.

#### 2.6.4. Validation of the Key Genes by RT-qPCR

RT-qPCR was employed to confirm the precision of the transcriptomic data through the examination of 9 distinct genes (details of gene names and corresponding primers were provided in Table S1). cDNA synthesis was carried out according to the protocol supplied with the PrimeScript RT reagent Kit combined with the gDNA Eraser kit (Takara Bio, Dalian, China). Subsequently, the RT-qPCR experiments were conducted using the TB Green^®^ Premix Ex Taq™ kit (Takara Bio, Dalian, China) on a CFX Connect PCR system (Bio-Rad Laboratories Co., Ltd., Hercules, CA, USA). The *ACT1* gene served as the reference. The comparative quantification of gene expression was determined by the 2^−ΔΔCt^ analysis method [19].

### 2.7. Statistical Analysis

All experiments were independently repeated three times under the same conditions, and the results are reported as mean ± standard deviation. Statistical analysis was performed using SPSS version 19.0 (*p* < 0.05) to determine the significance, while data representation was accomplished with Origin 2018.

## 3. Results

### 3.1. Growth Curve, Reducing Sugar Content, and Ethanol Content of S. Cerevisiae Under Lactic Acid Stress

**Figure 1:**
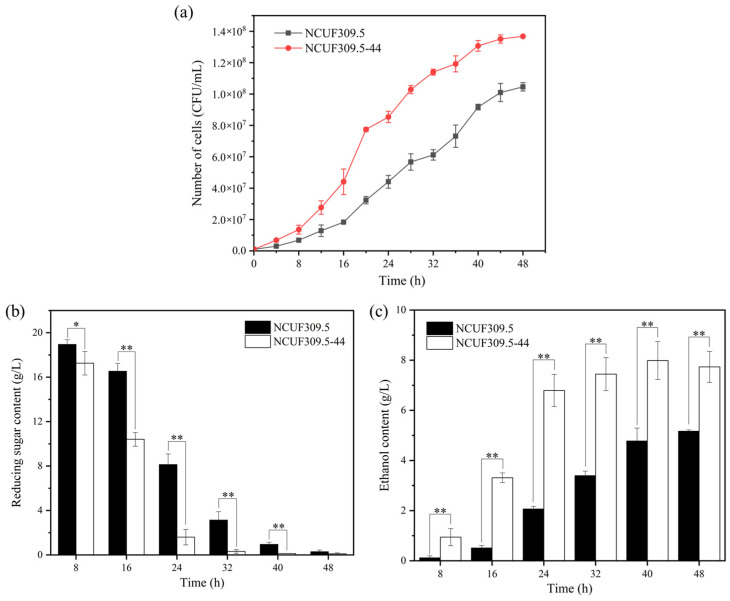
Effect of 4% lactic acid on growth curves (**a**), reducing sugar content (**b**), and ethanol content (**c**) of original and lactic acid-tolerant strains (* represents *p* < 0.05, ** represents *p* < 0.01).

The growth curves of *S. cerevisiae* NCUF309.5-44 and the original strain NCUF309.5 under 4% lactic acid were depicted in Figure 1a. As the incubation time increased, the cell number of both strains also increased. However, the cell number of *S. cerevisiae* NCUF309.5-44 was significantly higher than that of *S. cerevisiae* NCUF309.5. After 16 h of cultivation, the cell counts of the lactic acid-tolerant strain increased by approximately 2.41 times compared to the original strain, indicating that *S. cerevisiae* NCUF309.5-44 was indeed superior to the original strain. Additionally, the utilization of reducing sugars and the ethanol production capacity of the *S. cerevisiae* NCUF309.5-44 were significantly enhanced (Figure 1b,c). The ethanol content of the lactic acid-tolerant strain was 6.43 times higher than the original strain at 16 h, indicating that *S. cerevisiae* NCUF309.5-44 could elevate sugar metabolism efficiency to produce more energy in response to lactic acid stress, and it was capable of producing more ethanol. These results demonstrated the application prospects of *S. cerevisiae* NCUF309.5-44 in *Jiupei* with high concentration lactic acid and its potential for industrial application in *Baijiu* production. Further optimization and scale-up trials are ongoing to fully harness the potential of *S. cerevisiae* NCUF309.5-44 for commercial *Baijiu* production.

### 3.2. ROS Content of S. cerevisiae Under Lactic Acid Stress

**Figure 2:**
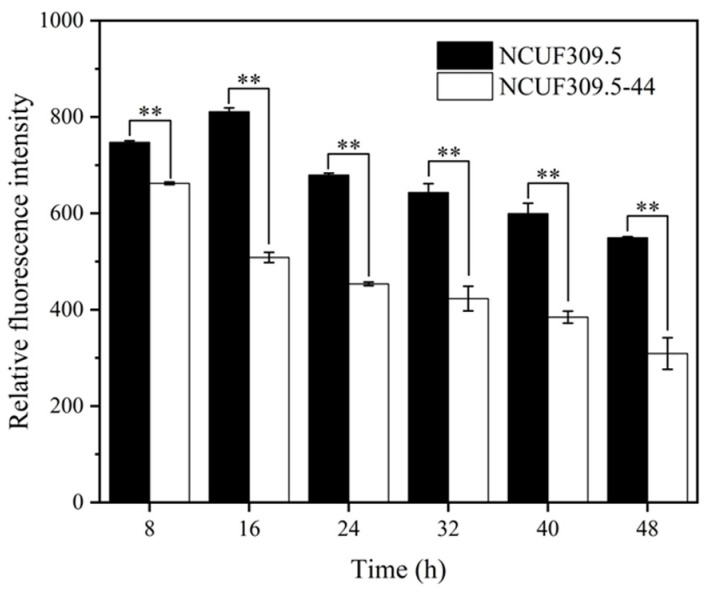
Effect of 4% lactic acid on ROS content of original and lactic acid-tolerant strains (** represents *p* < 0.01).

ROS are byproducts generated by yeast cells during their own aerobic metabolism when a portion of the oxygen cannot be completely reduced. Once faced with various external stress factors, the concentration of ROS increases, which may then damage key biomolecules in yeast cells, including proteins, DNA, and lipids, leading to cellular damage [20]. To investigate the extent of oxidative damage in *S. cerevisiae* NCUF309.5-44 and the original strain under 4% lactic acid stress, the cellular ROS content was measured (Figure 2). As time progressed, the ROS content produced by both strains showed a slow declining trend. However, throughout the entire cultivation process, the ROS content in *S. cerevisiae* NCUF309.5-44 remained significantly lower than that in the original strain. After 16 h of lactic acid stress, the ROS content in the lactic acid-tolerant strain was 37.29% lower than the original strain, indicating that it suffered from a relatively lighter degree of oxidative damage.

### 3.3. Antioxidant Enzyme Activity of S. cerevisiae Under Lactic Acid Stress

When confronted with oxidative stress caused by a burst of ROS, yeast cells can activate ROS scavenging mechanisms to mitigate the toxic effects. Among these, SOD is a key intracellular antioxidant enzyme that can convert the highly toxic superoxide anion into the less toxic H_2_O_2_. The CAT can further decompose H_2_O_2_ into water and oxygen, reducing the toxicity of ROS [21]. The GSH (glutathione)/GPx system is also a commonly existing ROS scavenging system within yeast cells. GPx is the key enzyme in the GSH/GPx system, and it plays an important role in removing excessive ROS [22]. The antioxidant enzyme activities of the lactic acid-tolerant and original strains under 4% lactic acid stress are listed in Table 1. The activities of the SOD, CAT, and GPx exhibited a similar trend, characterized by a gradual decline in enzyme activity as fermentation time was prolonged. This corresponded to the ROS results, where lower ROS levels were associated with lower antioxidant enzyme activity. The SOD and CAT activities of *S. cerevisiae* NCUF309.5-44 were significantly reduced at all time points compared to the original strain, while the GPx activity was significantly elevated, indicating that GPx played a key role in scavenging ROS.

### 3.4. Genomic Results

**Figure 3:**
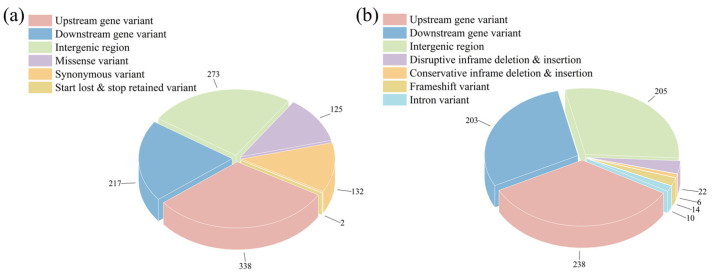
Results of different SNPs (**a**) and InDels (**b**) annotations between original and lactic acid-tolerant strains.

To further investigate the genomic variations that lead to the phenotypic changes, the whole-genome re-sequencing of the *S. cerevisiae* NCUF309.5-44 and *S. cerevisiae* NCUF309.5 was performed. As shown in Table S2, the Q30 base percentage was above 96.7% (generally required to be above 80%), and the GC content of the sequences was above 38.5%. By aligning the sequencing results with the known genome sequence of *S. cerevisiae* S228c, the percentage of reads for both yeast strains that corresponded to the reference genome accounted for 99.26% and 99.27% of the total reads, respectively. The percentage of reads that were paired-end and mapped to the genome with a distance consistent with the sequencing fragment length was 97.98% and 97.95%, respectively, indicating a high alignment efficiency and suggesting that the sample sequencing data were accurate. After filtering, 1785 mutation sites in *S. cerevisiae* NCUF309.5-44 compared to the original strain were found, including 1087 SNPs and 698 InDels. Annotation analysis of the mutation sites revealed that most of the SNPs in *S. cerevisiae* NCUF309.5-44 were located in the exon regions that encode proteins, resulting in 125 missense mutations that could affect protein function (Figure 3). For instance, the *CYB2* gene encoding L-lactate dehydrogenase and the *PMA2* gene encoding H^+^-ATPase both underwent missense mutations, resulting in the substitution of isoleucine with threonine at position 18 and valine to phenylalanine at position 173, respectively (Table 2). Additionally, the analysis revealed 42 InDels within the exon regions of the genome, which resulted in various types of deletions or mutations that could potentially affect the function of the genes (Figure 3).

**Figure 4:**
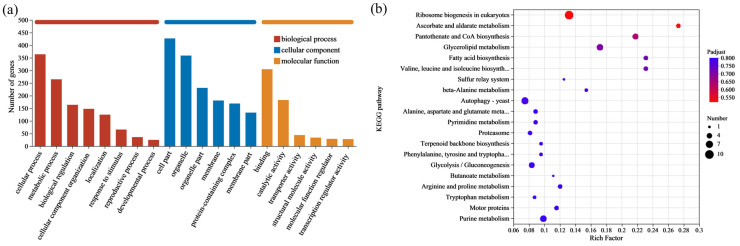
GO annotation (**a**) and KEGG enrichment (**b**) results of SNPs and InDels between original and lactic acid-tolerant strains.

GO is a system used to classify gene functions. When describing gene functions, the system considers the following aspects: Biological Process (BP), Molecular Function (MF), and Cellular Component (CC). These SNPs and InDels were compared to the GO database to investigate the potential impacts of the variations. The results indicated that the mutated genes were primarily concentrated in the following areas: cellular process and metabolic process in BP; cell part and organelle in CC; and binding and catalytic activity in MF. This suggested that genes related to these functions might have a significant impact on the lactic acid tolerance of the *S. cerevisiae* NCUF309.5-44 (Figure 4a).

Microorganisms often do not perform different biological functions due to the action of a single gene, but rather through the coordination of multiple genes. When studying and analyzing the pathways that encompassed the mutant genes, it is essential to perform enrichment significance testing. KEGG is the main public database for Pathways. By performing a BLAST+ 2.9.0 analysis of the obtained SNPs and InDels with the KEGG database, it was determined that 280 genes were annotated in different signaling pathways (Figure 4b). The statistical results indicated that the signal pathways with a larger number of annotated genes included ribosome biosynthesis in eukaryotes, glycerolipid metabolism, pantothenate CoA biosynthesis, and glycolysis/gluconeogenesis pathways. The KEGG analysis results revealed that metabolic pathways associated with ribosomes were significantly enriched. The main reason is that the ribosome serves as the site for protein synthesis within the cell in eukaryotes, where it reads the genetic information embedded within the mRNA nucleotide sequence and translates it into the sequence information of amino acids to synthesize proteins.

### 3.5. Transcriptomic Results

As shown in Table S3, the RNA concentrations extracted from the original strain and the lactic acid-tolerant strain far exceeded the experimental requirements for eukaryotic transcriptome libraries (35 ng/μL). Moreover, the OD_260/280_ values were all greater than 1.8, the OD_260/230_ values were all greater than 1.0, and the RIN (RNA Integrity Number, ranging from 1 to 10, with higher values indicating better RNA integrity) values were elevated. Therefore, it could be concluded that the RNA purity and integrity were high, and there was no contamination by proteins or other carbon sources, which met the standards for library construction.

To investigate the lactic acid tolerance mechanism of the lactic acid-tolerant strain at the transcriptomic level, transcriptomic sequencing was performed on the *S. cerevisiae* NCUF309.5 and *S. cerevisiae* NCUF309.5-44 after exposure to 4% lactic acid stress. As shown in Table S4, the mean error rate for the sequenced bases, as per the quality control metrics, was significantly below 0.1%., with a Q30 base percentage of over 96.32% (typically required to be over 80%), and the sequence GC content was above 41.87%. Sequence alignment results revealed that the matching rate of the sequences obtained from each sample with the target genome ranged from 95.47% to 97.28%, and the unique mapping rate ranged from 91.65% to 93.37%. This indicated that the sequencing data from the six samples in this study were reliable and suitable for further analysis.

**Figure 5:**
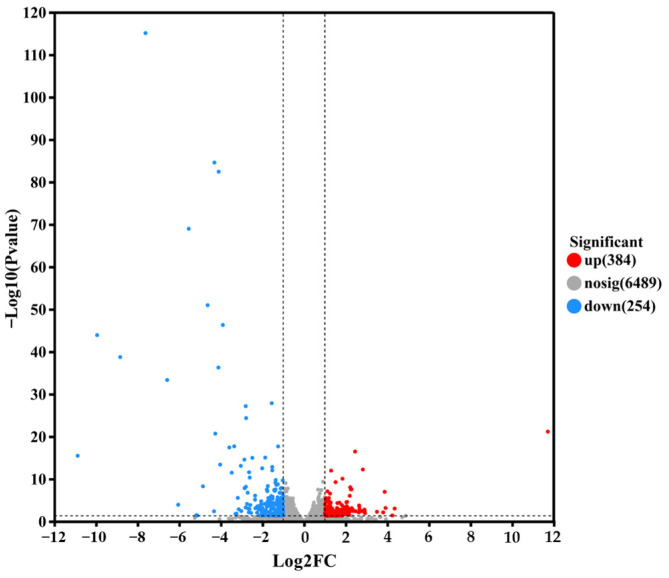
Volcanic map of DEGs between original and lactic acid-tolerant strains under lactic acid stress.

To further explore the lactic acid tolerance mechanism of the *S. cerevisiae* NCUF309.5-44, quantitative analysis of gene expression levels was conducted utilizing expression quantification software RSEM 1.3.3. The differential expression levels of genes between samples were calculated, and the number of DEGs was analyzed using the screening criteria of fold change (FC) ≥ 2 or ≤0.05 and *p* < 0.05 under 4% lactic acid concentrations. As shown in Figure 5, the lactic acid-tolerant strain exhibited 384 genes significantly upregulated and 254 genes significantly downregulated compared to the original strain under 4% lactic acid stress, indicating that there were differences in the biological metabolic reactions and regulatory mechanisms between the two strains under lactic acid stress. These differences in gene expression might contribute to the enhanced tolerance of *S. cerevisiae* NCUF309.5-44 to lactic acid.

In order to obtain the specific information and functions of the DEGs in *S. cerevisiae* under lactic acid stress, as well as to analyze their roles within the cell, GO functional annotation analysis was first conducted. The results indicated that the upregulated DEGs (up-DEGs) were primarily concentrated in the BP of cellular process, metabolic process, and biological regulation, in the CC of cell part, organelle, membrane, and in the MF of binding and catalytic activity (Figure S1a). On the other hand, the downregulated DEGs (down-DEGs) were mainly focused on BP, such as Cellular Component organization or biogenesis, CC-like organelle parts, and MF, such as structural molecular activity (Figure S1b).

**Figure 6:**
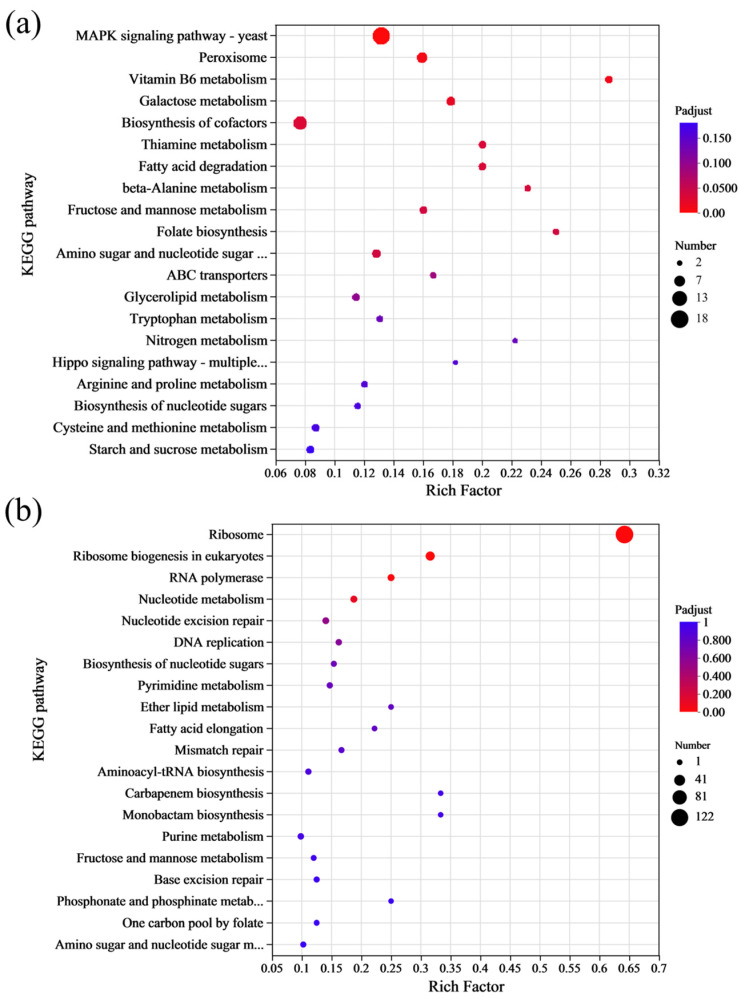
The results of KEGG enrichment analysis of DEGs between original and lactic acid-tolerant strains under lactic acid stress ((**a**): up-DEGs; (**b**): down-DEGs).

KEGG enrichment analysis was also carried out on the DEGs of *S. cerevisiae* under 4% lactic acid stress. The up-DEGs were primarily enriched in the yeast MAPK signaling pathway, peroxisome, and biosynthesis of cofactor pathways (Figure 6a), suggesting that these metabolic pathways may be key mechanisms for lactic acid tolerance in the *S. cerevisiae* NCUF309.5-44. In contrast, the down-DEGs were mainly enriched in pathways related to the ribosome, ribosome biogenesis in eukaryotic, and RNA polymerase (Figure 6b). The KEGG enrichment analysis indicated that acclimating to lactic acid stress involves a complex interplay of various cellular pathways, with some pathways being upregulated to cope with the stress and others being downregulated, potentially as a way to reallocate cellular resources or to reduce energy-consuming processes.

From the transcriptomic sequencing results, five up-DEGs and four down-DEGs were randomly selected, and the relative expression levels of these genes were measured using RT-qPCR methods. As depicted in Figure S2, the expression levels of the nine genes aligned with those noted in the transcriptomic data (Table S5), indicating that the transcriptomic data were accurate and reliable.

## 4. Discussion

*S. cerevisiae* is the most important microorganism in the Chinese solid-state *Baijiu* fermentation process. During fermentation, it is inhibited by high concentrations of lactic acid, leading to reduced growth and metabolism rates, ultimately affecting the production cycle and product quality of *Baijiu* [2]. In previous studies, we obtained a strain of *S. cerevisiae* NCUF309.5-44, capable of tolerating high concentrations of lactic acid, which showed significantly better growth under 4% lactic acid stress than the original strain (Figure 1a). Following the determination of reducing sugar and ethanol content, it was found that the *S. cerevisiae* NCUF309.5-44 could increase the rate of glycolysis and produce more ethanol substrates (Figure 1b,c), indicating a significant improvement in its metabolic capacity under high lactic acid concentrations. This strain showed promising application prospects in the production of *Baijiu*.

When yeast cells are exposed to external environmental stress, the intracellular ROS levels surge, causing cellular damage. Studies have shown that excellent stress-tolerant yeast strains can reduce ROS levels to increase cell survival rates in acidic environments [23,24]. This finding was aligned with our results, where the lactic acid-tolerant strain NCUF309.5-44 had significantly lower ROS levels than the original strain under lactic acid stress, showing a 37.29% reduction in ROS levels after 16 h of lactic acid exposure. This suggested that *S. cerevisiae* NCUF309.5-44 experienced less oxidative damage and could grow better under lactic acid stress (Figure 2). However, when we further investigated the antioxidant enzyme activities of the two strains, we observed that the activities of SOD and CAT in *S. cerevisiae* NCUF309.5-44 were significantly lower than the original strain, while the activity of GPx was notably elevated. This indicated that the strain might primarily rely on peroxidases such as GPx to scavenge ROS within the cell. The transcriptomic results further validated this finding; the expression levels of *SOD1*, *SOD2*, and *CTT1* genes significantly decreased in *S. cerevisiae* NCUF309.5-44, while the expression levels of *GSH1* and *GPX2* increased in contrast to *S. cerevisiae* NCUF309.5. A previous study confirmed that the GSH content in gene-edited acid-tolerant *S. cerevisiae* increased significantly under lactic acid stress, suggesting that the GPx-catalyzed reaction of GSH with peroxides to produce non-toxic hydroxyl compounds may have been a more effective strategy for scavenging ROS under lactic acid stress [25]. The lactic acid-tolerant strain in this study might have evolved more refined antioxidant strategies, choosing to rely heavily on GPx to clear ROS accumulated under lactic acid stress. In contrast, the original strain might have relied more on a more general antioxidant system (SOD/CAT) to cope with the general increase in ROS, and the efficiency of the SOD/CAT system might also have been inherently lower in the lactic acid environment, indicating weaker adaptability of original strain to lactic acid stress. Additionally, the research results of Li et al. [26] also showed that *S. cerevisiae* primarily initiated a GSH-mediated ROS clearance mechanism to deal with furfural stress.

**Figure 7:**
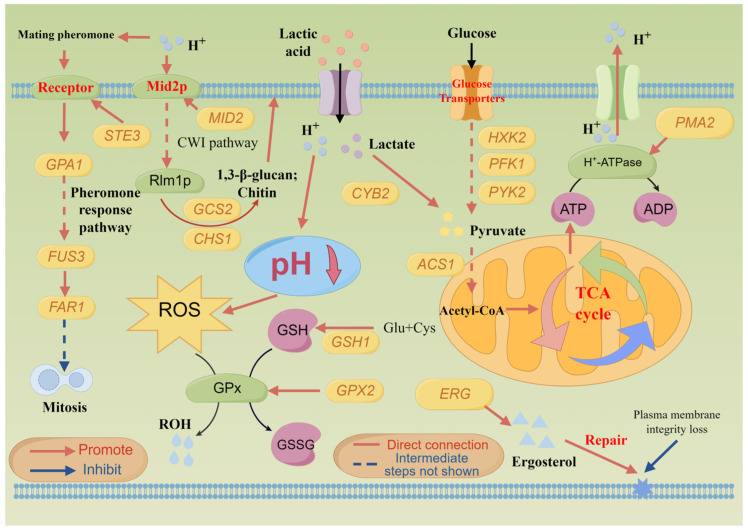
Schematic diagram of lactic acid tolerance mechanism of *S. cerevisiae* NCUF309.5-44.

Through a combined analysis of whole-genome re-sequencing and transcriptomic, it was found that the lactic acid tolerance mechanism of *S. cerevisiae* NCUF309.5-44 was related to multiple metabolic pathways. Firstly, *S. cerevisiae* NCUF309.5-44 responded to lactic acid stress by activating the MAPK signaling pathway, which was the most prominently upregulated pathway in the *S. cerevisiae* NCUF309.5-44 (Table S6). In the pheromone response pathway, *MFAL1* and *STE3* encoding the pheromone receptor were found to be increased by 9.94-fold and 8.83-fold, respectively, compared to the original strain. Upon binding of pheromone, Ste3p occurred a conformational change, leading to the exchange of GDP for GTP in the associated G protein alpha subunit (Gpa1), followed by the release of the subunit complex, which further activated downstream components of the protein [27]. After Fus3 activation, it could inhibit the cyclin by phosphorylating Far1, thereby arresting the yeast cell cycle at the G1 phase [28]. All these suggested that *S. cerevisiae* NCUF309.5-44 might affect the cell cycle under lactic acid stress, preventing the cell from entering mitosis. The stagnation of the cell cycle might help the cell conserve energy and repair damage, thereby enhancing acid tolerance (Figure 7). The down-DEGs were enriched in the DNA replication pathway in KEGG enrichment also supported this result. Additionally, Ste12p, encoded by the *STE12*, was an important transcription factor in *S. cerevisiae*. Dig1 (encoded by the *DIG1*) served as a negative regulator of Ste12p activity. When the yeast cells received the mating pheromone signal, Dig1 was phosphorylated, releasing Ste12p, allowing it to activate the expression of mating-related genes (such as *FAR1*, *STE5*), thereby enabling the cells to respond to external stimuli [29]. Mid2p, encoded by *MID2*, is a sensor protein on the cell wall and a key receptor in the CWI pathway. The expression level of *MID2* was notably upregulated in *S. cerevisiae* NCUF309.5-44, indicating that the CWI pathway was activated by Mid2p under lactic acid stress. In addition, two SNP mutations were found in the *MID2* gene of *S. cerevisiae* NCUF309.5-44, illustrating that it was important for lactic acid tolerance in yeast. Studies have reported that the deletion of *MID2* could result in a decrease in the growth performance of strains under acetic acid stress [30]. Moreover, the expression of *RLM1*, a transcription factor downstream of Mid2p, was also upregulated in *S. cerevisiae* NCUF309.5-44. The stimulation of Rlm1p enhanced the transcription of genes related to the synthesis and repair of cell wall components, such as *NCW2* (1.54-fold, involved in maintaining the balance of glucan/chitin) [31], *GSC2* (1.66-fold, encoding 1,3-β-glucan synthase), and *CHS1* (1.00-fold, encoding chitin synthase) in *S. cerevisiae* NCUF309.5-44. These helped the cell resist external pressures and maintain the normal morphology and physiological functions of *S. cerevisiae* NCUF309.5-44 cells under lactic acid stress. As previously reported in our studies, the cell integrity of *S. cerevisiae* NCUF309.5-44 under lactic acid stress was maintained much better than the original strain [4]. The upregulation of these genes and the activation of the CWI pathway suggested that *S. cerevisiae* NCUF309.5-44 adapted to lactic acid stress by reinforcing its cell wall, which was essential for maintaining cell integrity and protecting against the toxic effects of lactic acid (Figure 7). Previous studies demonstrated that when yeast cells were exposed to inhibitory concentrations of weak acids, the yeast CWI pathway was activated, leading to a corresponding thickening of the cell wall to reduce its porosity, thereby decreasing the influx of undissociated acids [32].

Secondly, *S. cerevisiae* NCUF309.5-44 responded to lactic acid stress by adjusting the ribosome pathway. The ribosome serves as the location for protein synthesis, and nearly all proteins within a cell are synthesized by ribosomes. Normal ribosome synthesis is fundamental to the healthy growth of yeast cells. RNA polymerases I, II, and III work together in the process of ribosome biogenesis, producing the various components needed for ribosome assembly. Only when these components are correctly assembled can the ribosome perform its function in protein synthesis, thereby participating in various life activities of yeast cells, such as cell metabolism, growth, and reproduction [13]. Studies have found that acid stress initiated a response mechanism in yeast, leading to the upregulation of genes involved in pathways related to ribosome biogenesis in eukaryotes, as well as RNA polymerases [33,34]. While *S. cerevisiae* NCUF309.5-44 showed the downregulation of genes in these pathways compared to *S. cerevisiae* NCUF309.5 in this study (Table S6), reflecting an improved tolerance to lactic acid in the lactic acid-tolerant strain. Similarly, Yang et al. [35] also found that the expression of most genes related to ribosomes was downregulated after transcriptomic analysis of an acid-tolerant *S. cerevisiae* strain.

Thirdly, *S. cerevisiae* NCUF309.5-44 responded to lactic acid stress by upregulating glycolysis/gluconeogenesis metabolism, which is a crucial energy metabolic pathway in *S. cerevisiae* [36]. The transcriptomic findings revealed that genes in the glycolysis pathway of *S. cerevisiae* NCUF309.5-44, including *HXK2*, *ENO1*, *PFK1*, *PYK2*, *PDC1*, *ALD4*, *ALD6*, *ADH5*, *ADH6*, and *ADH7* genes, were significantly upregulated compared to *S. cerevisiae* NCUF309.5, suggesting that *S. cerevisiae* NCUF309.5-44 could generate more energy by enhancing the glycolytic pathway in response to lactic acid stress (Figure 7). This also explained the results in Figure 1b,c, where the utilization rate of reducing sugar and the production rate of ethanol in *S. cerevisiae* NCUF309.5-44 under 4% lactic acid stress was significantly higher than the original strain. The genomic results revealed that the *CYB2* gene, which encodes lactate dehydrogenase in *S. cerevisiae* NCUF309.5-44, had undergone a missense mutation (Table 2). The expression level of *CYB2* in the transcriptome is 1.55 times higher than that of the original strain, indicating that *S. cerevisiae* NCUF309.5-44 enhanced the ability to metabolize lactic acid, which allowed it to reduce lactic acid concentration more quickly and alleviate stress intensity. Additionally, the *ACS1* gene encoding acetyl-CoA synthetase had also undergone a variation, and its expression level was significantly increased by 2.55 times in *S. cerevisiae* NCUF309.5-44, suggesting that after lactic acid entered the glycolysis pathway, the content of pyruvate was increased, which in turn promoted the synthesis of acetyl-CoA. Indeed, acetyl-CoA is a pivotal compound that enters the TCA cycle, where it is further oxidized to release a large amount of energy [37]. The transcriptomic data also revealed that the expression of the *CIT2* gene encoding citrate synthase, the enzyme responsible for facilitating the condensation of acetyl-CoA with oxaloacetate to produce citrate, was increased by 1.31 times in *S. cerevisiae* NCUF309.5-44. This proved that the TCA cycle in *S. cerevisiae* NCUF309.5-44 was enhanced, enabling the generation of additional ATP to counteract environmental challenges in comparison with *S. cerevisiae* NCUF309.5. What is more, the increased flux through the TCA cycle could provide the necessary intermediates for other biosynthetic pathways, which is essential for the anaplerotic reactions that sustain cell metabolism under lactic acid stress. In addition, the *PYC1* gene encoding pyruvate carboxylase was significantly upregulated by 1.86 times, and SNP mutation was also found in this gene, indicating that *S. cerevisiae* NCUF309.5-44 could further strengthen the TCA cycle by increasing the conversion rate of pyruvate to oxaloacetic acid.

In addition to the aforementioned metabolic pathways, several other genes also played important roles in the lactic acid tolerance of *S. cerevisiae* NCUF309.5-44. The upregulation of genes involved in ergosterol biosynthesis compared to *S. cerevisiae* NCUF309.5 was observed, including *ERG1* (1.32 times), *ERG2* (1.46 times), *ERG3* (1.33 times), *ERG5* (1.38 times), and *ERG8* (1.57 times), with *ERG8* also finding a missense mutation in the genome in *S. cerevisiae* NCUF309.5-44 (Table 2). Ergosterol was the main sterol found in yeast cells and was vital for maintaining the normal function of the cell membrane [11]. The accumulation of ergosterol contributed to protecting the cell membrane from acid stress, thereby enhancing the acid resistance of yeast cells [12]. Furthermore, multiple genes involved in ergosterol biosynthesis have been identified as determining factors for acquired tolerance or as exhibiting transcriptional responses to acid stress [38].

Finally, *S. cerevisiae* NCUF309.5-44 enhanced lactic acid tolerance by activating proton efflux. The plasma membrane H^+^-ATPase is recognized as a pivotal element for extrusion efflux in yeast, primarily driving the expulsion of H^+^ from the cell by hydrolyzing ATP [39]. However, the *PMA1* gene, which encodes the plasma membrane H^+^-ATPase, exhibited decreased expression levels in *S. cerevisiae* NCUF309.5-44 compared to the original strain. Interestingly, the *PMA2* gene, which belongs to the plasma membrane H⁺-ATPase gene family along with the *PMA1* gene, was found to undergo a missense mutation (Table 2), and transcriptomic analysis revealed its expression level was 2.00 times higher than *S. cerevisiae* NCUF309.5. This indicated that *S. cerevisiae* NCUF309.5-44 developed a mechanism to compensate for the downregulation of the *PMA1* gene by enhancing the expression of the *PMA2* gene, and, finally, achieved extruded protons and maintained intracellular Ph (Figure 7). The increased expression and potential functional changes in the *PMA2* gene due to the missense mutation could be a significant factor in the strain’s ability to tolerate lactic acid stress.

## 5. Conclusions

This study revealed the lactic acid tolerance mechanism of *S. cerevisiae* by measuring the growth, metabolic performance, and antioxidant enzyme activity, as well as identifying genetic variations and differentially expressed genes between the *S. cerevisiae* NCUF309.5-44 and *S. cerevisiae* NCUF309.5 through whole-genome re-sequencing and transcriptomic analyses under 4% (*v*/*v*) lactic acid stress. The results demonstrated that *S. cerevisiae* NCUF309.5-44 suffered less growth impact, produced higher ethanol content, and activated the GSH/GPx system, resulting in lower intracellular ROS content under lactic acid stress. Furthermore, *S. cerevisiae* NCUF309.5-44 responded to lactic acid stress by activating the pheromone response pathway and the cell wall integrity pathway. Meanwhile, the capacity of strains to maintain the cell membrane and proton extrusion was strengthened. Additionally, its glycolysis/gluconeogenesis metabolism was also enhanced. In brief, this study clarified the lactic acid tolerance mechanisms of *S. cerevisiae* NCUF309.5-44. It is anticipated that this research will furnish a foundation for using the lactic acid-tolerant *S. cerevisiae* NCUF309.5-44 in the production of Chinese solid-state *Baijiu* to improve its yield and quality. Additionally, it provides a conceptual basis for developing lactic acid-tolerant genetically engineered yeast strains, contributing to the creation of robust strains for bioethanol production.

## Figures and Tables

**Table 1:** Effects of 4% lactic acid on antioxidant enzyme activities of original and lactic acid-tolerant strains.

	Groups	Time (h)					
		8	16	24	32	40	48
SOD (U/g)	NCUF309.5	26.67 ± 0.32	27.25 ± 0.33	26.38 ± 0.67	24.97 ± 0.97	24.28 ± 0.60	23.14 ± 0.53
	NCUF309.5-44	19.57 ± 0.61 **	18.71 ± 0.37 **	17.56 ± 0.62 **	16.21 ± 0.35 **	14.38 ± 0.36 **	14.20 ± 0.62 **
CAT (U/g)	NCUF309.5	16.63 ± 0.42	17.23 ± 0.38	16.72 ± 0.74	16.16 ± 0.31	15.78 ± 0.59	15.21 ± 0.22
	NCUF309.5-44	9.60 ± 0.39 **	9.86 ± 0.57 **	9.26 ± 0.44 **	9.06 ± 0.21 **	8.71 ± 0.08 **	8.32 ± 0.14 **
GPx (U/g)	NCUF309.5	0.64 ± 0.02	0.59 ± 0.04	0.45 ± 0.06	0.41 ± 0.03	0.37 ± 0.01	0.32 ± 0.07
	NCUF309.5-44	0.94 ± 0.02 **	1.05 ± 0.10 **	0.89 ± 0.15 **	0.80 ± 0.09 **	0.70 ± 0.04 **	0.66 ± 0.03 **

All values are reported as the mean (±SD) of three experiments. ** represents significant differences vs. NCUF309.5 (*p* < 0.01).

**Table 2:** Some mutations present between original and lactic acid-tolerant strains.

Position	Gene	Type	Reference	Allele	Protein Change	Description
45019	*ACS1*	SNPs	A	G	Ser2Pro	Acetyl-CoA synthetase
175314	*TIR1*	SNPs	C	G	Gln23Glu	GPI-anchored mannoprotein
167729	*CYB2*	SNPs	A	G	Ile18Thr	L-lactate dehydrogenase
712385	*ERG8*	SNPs	A	C	Thr24Pro	Ergosterol synthesis
483359	*PMA2*	SNPs	G	T	Val173Phe	H^+^-ATPase
207682	*FLO1*	InDels	C	CAACTATCAATACTG	Ile1428fs	Cell wall protein

## Data Availability

The original contributions presented in this study are included in the article/Supplementary Materials. Further inquiries can be directed to the corresponding author.

## Supplementary Materials

The following supporting information can be downloaded at: https://www.mdpi.com/article/10.3390/foods14122027/s1, Figure S1: GO annotation analysis of differentially expressed genes between original and lactic acid-tolerant strains under lactic acid stress (a: up-DEGs; b: down-DEGs); Figure S2: verification of transcriptome results by qPCR; Table S1: list of primers used in real-time PCR analysis; Table S2: sequencing quality statistics; Table S3: results detected by Nanodrop for RNA; Table S4: statistical analysis of the sequencing data; Table S5: transcriptomics sequencing results of the validated gene; Table S6: statistical table of partial DEGs in the KEGG co-enrichment pathway.

## Abbreviations

The following abbreviations are used in this manuscript:

ARTP	Atmospheric and Room Temperature Plasma
MMC	Microbial Microdroplet Culture
NCBI	National Center for Biotechnology Information
YPD	Yeast extract peptone dextrose
ROS	Reactive oxygen species
SOD	Superoxide dismutase
CAT	Catalase
GPx	Glutathione peroxidase
PBS	Phosphate-buffered saline
DEGs	Differentially expressed genes
SNP	Single-nucleotide polymorphisms
InDels	Insertions/deletions
FC	Fold change
GO	Gene Ontology
KEGG	Kyoto Encyclopedia of Genes and Genomes
RT-qPCR	Quantitative real-time polymerase chain reaction
MAPK	Mitogen-activated protein kinase
GSH	Glutathione

## References

[1] Li K., Chen Y., Liu T., Deng M., Xu Z., Fu G., Wan Y., Chen F., Zheng F. (2020). Analysis of spatial distribution of bacterial community associated with accumulation of volatile compounds in *Jiupei* during the brewing of special-flavor liquor. LWT-Food Sci. Technol..

[2] Chen Y.R., Yang Y.L., Cai W.Q., Zeng J.L., Liu N., Wan Y., Fu G.M. (2023). Research progress of anti-environmental factor stress mechanism and anti-stress tolerance way of *Saccharomyces cerevisiae* during the brewing process. Crit. Rev. Food Sci. Nutr..

[3] Deng N., Du H., Xu Y. (2020). Cooperative response of *Pichia kudriavzevii* and *Saccharomyces cerevisiae* to lactic acid stress in *Baijiu* fermentation. J. Agric. Food Chem..

[4] Fan H.W., Wan Y., Huang Y.X., Yuan J.Y., Fan J.H., Kou Y.R., Yu X.F., Pan Y.F., Huang D., Fu G.M. (2025). Breeding of lactic acid-tolerant *Saccharomyces cerevisiae* based on atmospheric and room temperature plasma technology and automatic high-throughput microbial microdroplet culture system. Food Microbiol..

[5] Zeng L.J., Si Z.Y., Zhao X.M., Feng P.X., Huang J.X., Long X.F., Yi Y. (2022). Metabolome analysis of the response and tolerance mechanisms of *Saccharomyces cerevisiae* to formic acid stress. Int. J. Biochem. Cell Biol..

[6] Ribeiro R.A., Vitorino M.V., Godinho C.P., Bourbon-Melo N., Robalo T.T., Fernandes F., Rodrigues M.S., Sá-Correia I. (2021). Yeast adaptive response to acetic acid stress involves structural alterations and increased stiffness of the cell wall. Sci. Rep..

[7] Li B., Xie C.Y., Yang B.X., Gou M., Xia Z.Y., Sun Z.Y., Tang Y.Q. (2020). The response mechanisms of industrial *Saccharomyces cerevisiae* to acetic acid and formic acid during mixed glucose and xylose fermentation. Process Biochem..

[8] Hakkaart X., Liu Y.Y., Hulst M., el Masoudi A., Peuscher E., Pronk J., van Gulik W., Daran-Lapujade P. (2020). Physiological responses of *Saccharomyces cerevisiae* to industrially relevant conditions: Slow growth, low pH, and high CO_2_ levels. Biotechnol. Bioeng..

[9] Chen Y.R., Wan Y., Cai W.Q., Che X.M., Sun X.H., Peng H., Luo H.B., Huang D., Fu G.M. (2023). Transcriptomic and metabonomic to evaluate the effect mechanisms of the growth and aroma-producing of *Pichia anomala* under ethanol stress. Food Biosci..

[10] Holyavkin C., Turanli-Yildiz B., Yilmaz Ü., Alkim C., Arslan M., Topaloglu A., Kisakesen H.I., de Billerbeck G., François J.M., Çakar Z.P. (2023). Genomic, transcriptomic, and metabolic characterization of 2-Phenylethanol-resistant *Saccharomyces cerevisiae* obtained by evolutionary engineering. Front. Microbiol..

[11] Fletcher E., Feizi A., Bisschops M.M.M., Hallström B.M., Khoomrung S., Siewers V., Nielsen J. (2017). Evolutionary engineering reveals divergent paths when yeast is adapted to different acidic environments. Metab. Eng..

[12] Tian T.T., Wu D.H., Ng C.T., Yang H., Sun J.Y., Liu J.M., Lu J. (2020). Uncovering mechanisms of greengage wine fermentation against acidic stress via genomic, transcriptomic, and metabolic analyses of *Saccharomyces cerevisiae*. Appl. Microbiol. Biotechnol..

[13] Li M., Deng M., Chen Y., Fan H., Huang Y., Huang Y., Wan Y., Fu G. (2023). Exploring the stress mechanism of tannic acid on *Saccharomyces cerevisiae* based on transcriptomics. Food Biosci..

[14] Yang W., Liu S., Marsol-Vall A., Tahti R., Laaksonen O., Karhu S., Yang B., Ma X. (2021). Chemical composition, sensory profile and antioxidant capacity of low-alcohol strawberry beverages fermented with *Saccharomyces cerevisiae* and *Torulaspora delbrueckii*. LWT-Food Sci. Technol..

[15] Kang X., Gao Z.H., Zheng L.J., Zhang X.R., Li H. (2021). Regulation of Lactobacillus plantarum on the reactive oxygen species related metabolisms of *Saccharomyces cerevisiae*. LWT-Food Sci. Technol..

[16] Chen Y., Wan Y., Cai W., Liu N., Zeng J., Liu C., Peng H., Fu G. (2022). Effects on cell membrane integrity of *Pichia anomal* by the accumulating excessive reactive oxygen species under ethanol stress. Foods.

[17] Chen S., Zhou Y., Chen Y., Gu J. (2018). Fastp: An ultra-fast all-in-one FASTQ preprocessor. Bioinformatics.

[18] Jung Y., Han D. (2022). BWA-MEME: BWA-MEM emulated with a machine learning approach. Bioinformatics.

[19] Cai W.Q., Wan Y., Chen Y.R., Fan H.W., Li M.X., Wu S.W., Lin P., Zeng T.T., Luo H.B., Huang D. (2024). Transcriptomics to evaluate the influence mechanisms of ethanol on the ester production of *Wickerhamomyces anomalus* with the induction of lactic acid. Food Microbiol..

[20] Câmara A.D., Maréchal P.A., Tourdot-Maréchal R., Husson F. (2019). Oxidative stress resistance during dehydration of three non-*Saccharomyces* wine yeast strains. Food Res. Int..

[21] Wang G., Zhang T., Sun W., Wang H., Yin F., Wang Z., Zuo D., Sun M., Zhou Z., Lin B. (2017). Arsenic sulfide induces apoptosis and autophagy through the activation of ROS/JNK and suppression of Akt/mTOR signaling pathways in osteosarcoma. Free Radic. Biol. Med..

[22] Kontogianni V.G., Tsiafoulis C.G., Roussis I.G., Gerothanassis I.P. (2017). Selective 1D TOCSY NMR method for the determination of glutathione in white wine. Anal. Methods.

[23] Tian T., Wu D., Ng C.-T., Yang H., Sun J., Liu J., Lu J. (2020). A multiple-step strategy for screening *Saccharomyces cerevisiae* strains with improved acid tolerance and aroma profiles. Appl. Microbiol. Biotechnol..

[24] Saengphing T., Sattayawat P., Kalawil T., Suwannarach N., Kumla J., Yamada M., Panbangred W., Rodrussamee N. (2024). Improving furfural tolerance in a xylose-fermenting yeast *Spathaspora passalidarum* CMUWF1-2 via adaptive laboratory evolution. Microb. Cell Fact..

[25] Nugroho R.H., Yoshikawa K., Shimizu H. (2015). Metabolomic analysis of acid stress response in *Saccharomyces cerevisiae*. J. Biosci. Bioeng..

[26] Li Q., Zhang Z.Y., Ayepa E., Xiang Q.J., Yu X.M., Zhao K., Zou L.K., Gu Y.F., Li X., Chen Q. (2023). Discovery of new strains for furfural degradation using adaptive laboratory evolution in *Saccharomyces cerevisiae*. J. Hazard. Mater..

[27] Herskowitz I. (1995). Map kinase pathways in yeast—For mating and more. Cell.

[28] Alberghina L., Rossi R.L., Querin L., Wanke V., Vanoni M. (2004). A cell sizer network involving Cln3 and Far1 controls entrance into S phase in the mitotic cycle of budding yeast. J. Cell Biol..

[29] Aristizabal M.J., Negri G.L., Kobor M.S. (2015). The RNAPII-CTD maintains genome integrity through inhibition of retrotransposon gene expression and transposition. PLoS Genet..

[30] Orij R., Urbanus M.L., Vizeacoumar F.J., Giaever G., Boone C., Nislow C., Brul S., Smits G.J. (2012). Genome-wide analysis of intracellular pH reveals quantitative control of cell division rate by pHc in *Saccharomyces cerevisiae*. Genome Biol..

[31] Queiroz M.G., Elsztein C., de Morais M.A. (2020). The effects of the NCW2 protein of *Saccharomyces cerevisiae* on the positioning of chitin in response to cell wall damage. Antonie Van Leeuwenhoek.

[32] Levin D.E. (2005). Cell wall integrity signaling in *Saccharomyces cerevisiae*. Microbiol. Mol. Biol. Rev..

[33] Zeng L.J., Huang J.X., Feng P.X., Zhao X.M., Si Z.Y., Long X.F., Cheng Q.W., Yi Y. (2022). Transcriptomic analysis of formic acid stress response in *Saccharomyces cerevisiae*. World J. Microbiol. Biotechnol..

[34] Wang N., Zhang P.Y., Zhou X.L., Zheng J., Ma Y., Liu C.G., Wu T., Li H., Wang X.Q., Wang H. (2023). Isolation, Identification, and Characterization of an Acid-Tolerant *Pichia kudriavzevii* and Exploration of Its Acetic Acid Tolerance Mechanism. Fermentation.

[35] Yang P.Z., Chen J.C., Wu W.J., Jiang S.W., Deng Y.H., Lu J.L., Wang H., Zhou Y., Geng Y.Y., Zheng Z. (2023). *Saccharomyces cerevisiae* MET5DeltaSIZ1Delta enhancing organic acid tolerance with *XYL1* and *XYL2* integration for ethanol yield improvement in the presence of xylose and low pH value. LWT-Food Sci. Technol..

[36] Overal G.B., Teusink B., Bruggeman F.J., Hulshof J., Planqué R. (2018). Understanding start-up problems in yeast glycolysis. Math. Biosci..

[37] Sakihama Y., Hidese R., Hasunuma T., Kondo A. (2019). Increased flux in acetyl-CoA synthetic pathway and TCA cycle of *Kluyveromyces marxianus* under respiratory conditions. Sci. Rep..

[38] Palma M., Guerreiro J.F., Sá-Correia I. (2018). Adaptive Response and Tolerance to Acetic Acid in *Saccharomyces cerevisiae* and *Zygosaccharomyces bailii*: A Physiological Genomics Perspective. Front. Microbiol..

[39] Ndukwe J.K., Aliyu G.O., Onwosi C.O., Chukwu K.O., Ezugworie F.N. (2020). Mechanisms of weak acid-induced stress tolerance in yeasts: Prospects for improved bioethanol production from lignocellulosic biomass. Process Biochem..

