# Supplementary material for: Deciphering the Molecular Adapting Mechanism of Lactic Acid-Tolerant Saccharomyces cerevisiae Through Genomic and Transcriptomic Analysis
Source: Foods. 2025 Jun 8;14(12):2027. doi: 10.3390/foods14122027 (PMC12191993; doi:10.3390/foods14122027)
Supplement: Supplementary file 1 [file foods-14-02027-s001.zip › foods-3666942-supplementary Figures.pdf]

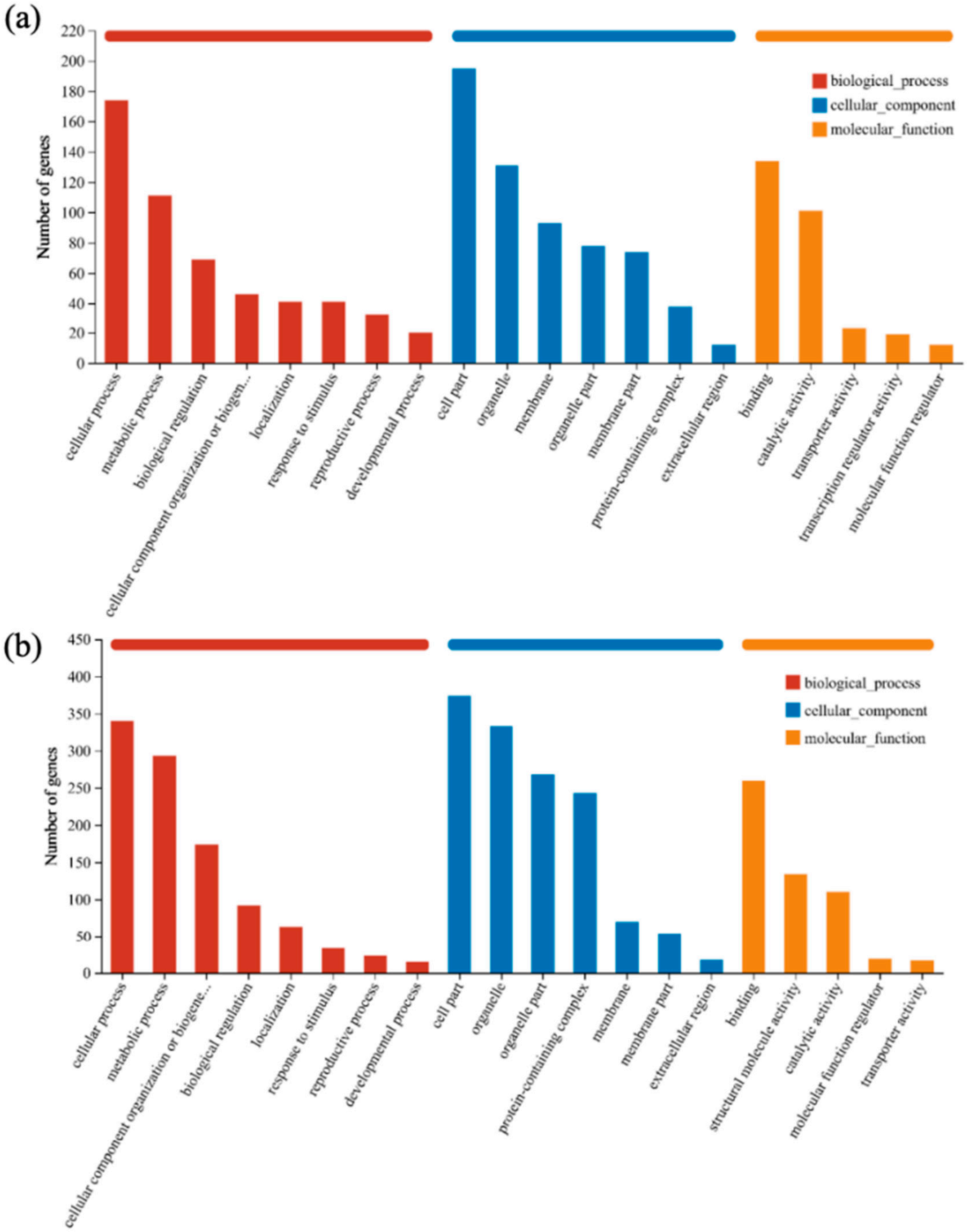

Figure S1 GO annotation analysis of differentially expressed genes between original and lactic acid-tolerant strains under lactic acid stress (a: up-DEGs; b: down-DEGs)

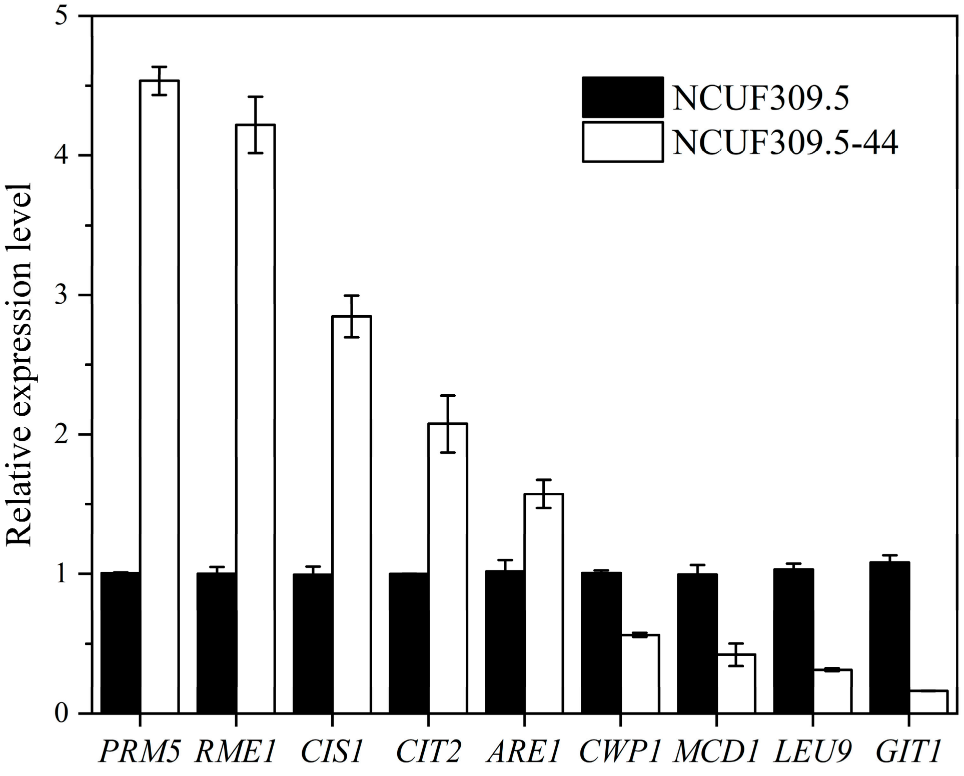

Figure S2 Verification of transcriptome results by qPCR
