# Supplementary material for: Deciphering the Molecular Adapting Mechanism of Lactic Acid-Tolerant Saccharomyces cerevisiae Through Genomic and Transcriptomic Analysis
Source: Foods. 2025 Jun 8;14(12):2027. doi: 10.3390/foods14122027 (PMC12191993; doi:10.3390/foods14122027)
Supplement: Supplementary file 1 [file foods-14-02027-s001.zip › foods-3666942-Supplementary Tables.pdf]

Table S1 List of primers used in real-time PCR analysis

Genes	Primer sequence Forward: 5′-3′	Primer sequence Reverse: 5′-3′
PRM5	GCCAAGAGAGGCCTTCCAAA	CGGACTCGCTGATATGGACC
RME1	CAAAACAGTGCCATCGCCAA	CACCATGGCCACATGTCTCT
CIS1	ACGTTGTTGGTTCAGGCAGA	CCTGGGCAGCCTTGAGTAAA
CIT2	AGAGGTATTCCAGGGAGCGT	ATCGGCATTTGGGTCCACTT
ARE1	ACTCAATTCCGCAGAAGCCA	GCTTATTCCATGCACTGCCG
CWP1	CCTGGTGAGTATCCGTTCCG	TGCCAAGTTTCAAAGTGCCG
MCD1	TACCAGTGGGCTTAATGGCG	TCAATTGACCCTTCTCGCCC
LEU9	GACCCAATGTCTGTGGCTCA	AGGACCAACGAGTAGCTTGC
GIT1	TGGTAGTGCTCTGTGTGCTG	TGGCCAGAAGCACCCTATTG
ACT1	GAAATGCAAACCGCTGCTCA	TACCGGCAGATTCCAAACCC

Table S2 Sequencing quality statistics

Sample	Raw reads	Raw bases (bp)	Clean reads	Clean GC (%)	Clean Q30 (%)	Mapped Ratio (%)	Proper Ratio (%)
NCUF 309.5	6,792,522	1,025,670,822	6,757,512	38.54	96.93	99.26	97.98
NCUF 309.5-44	7,216,752	1,089,729,552	7,173,992	38.58	96.72	99.27	97.95

Table S3 Results detected by Nanodrop for RNA

Samples	Concentration (ng/μL)	$OD_{260/280}$	$OD_{260/230}$	RIN
NCUF309.5-1	764.19	2.21	2.45	10
NCUF309.5-2	722.72	2.19	2.47	9.9
NCUF309.5-3	732.45	2.22	2.37	10
NCUF309.5-44-1	2107.87	2.21	2.53	10
NCUF309.5-44-2	2198.57	2.20	2.48	10
NCUF309.5-44-3	2524.55	2.21	2.53	9.7

Table S4 Statistical analysis of the sequencing data

Sample	Raw reads	Raw bases	Clean reads	Error rate (%)	Q30 (%)	GC content (%)	Total mapped	Uniquely mapped
NCUF309.5-1	46319578	6994256278	45965550	0.0117	96.54	41.87	44716330 (97.28%)	42564574 (92.6%)
NCUF309.5-2	41992984	6340940584	41692584	0.0118	96.32	41.97	40104983 (96.19%)	38632186 (92.66%)
NCUF309.5-3	44106688	6660109888	43781756	0.0117	96.49	42.02	42150312 (96.27%)	40877369 (93.37%)
NCUF309.5-44-1	46767740	7061928740	46430726	0.0117	96.5	41.93	44325367 (95.47%)	42612893 (91.78%)
NCUF309.5-44-2	42269334	6382669434	41998514	0.0117	96.52	41.96	40127750 (95.55%)	38490941 (91.65%)
NCUF309.5-44-3	47746048	7209653248	47436638	0.0117	96.53	42.1	45656850 (96.25%)	43822069 (92.38%)

Table S5 Transcriptomics sequencing results of the validated gene

Gene	Description	log_2 (Fold change)
PRM5	Pheromone-regulated protein	4.11
RME1	Zinc finger protein involved in control of meiosis	4.03
CIS1	Protein of unknown function found in mitochondria	2.71
CIT2	Citrate synthase	1.31
ARE1	Acyl-CoA:sterol acyltransferase	1.11
CWP1	Cell wall mannoprotein that localizes to birth scars of daughter cells	-2.07
MCD1	Essential alpha-kleisin subunit of the cohesin complex	-2.41
LEU9	Alpha-isopropylmalate synthase II	-2.67
GIT1	Plasma membrane permease	-3.36

Table S6 Statistical table of partial DEGs in the KEGG co-enrichment pathway

Pathway id	Gene name	Description	log2 (Fold change)
MAPK signaling pathway - yeast	*MFAL1*	mating pheromone alpha-factor	9.94
	STE3	pheromone a factor receptor	8.83
	SST2	GTPase-activating protein SST2	1.76
	GPA1	guanine nucleotide-binding protein alpha-1 subunit	4.31
	GNB1	guanine nucleotide-binding protein G(I)/G(S)/G(T) subunit beta-1	2.87
	GNG	guanine nucleotide-binding protein subunit gamma, fungi	6.58
	FAR1	cyclin-dependent kinase inhibitor FAR1	3.59
	STE5	pheromone-response scaffold protein	2.78
	MAPK1_3	mitogen-activated protein kinase 1/3	5.54
	STE12	transcription factor STE12	1.57
	DIG1	down-regulator of invasive growth 1	1.25
	MID2	mating pheromone-induced death protein 2	1.02
	MIH1	M-phase inducer tyrosine phosphatase	1.00
	MSS11	transcription activator MSS11	1.27
	TEAD	transcriptional enhancer factor	2.80
	FLO11	flocculation protein FLO11	3.03
Peroxisome	*ACOX1*	acyl-CoA oxidase	1.37
	PXA1	ATP-binding cassette, subfamily D (ALD), peroxisomal long-chain fatty	1.97

		acid import protein	
	FAA2	long-chain acyl-CoA synthetase	1.84
	ECI2	Delta3-Delta2-enoyl-CoA isomerase	1.29
	DCI1	Delta3,5-Delta2,4-dienoyl-CoA isomerase	1.51
	IDH1	isocitrate dehydrogenase	1.05
Biosynthesis of cofactors	*ALDH*	aldehyde dehydrogenase (NAD+)	2.18
	pdxT	pyridoxal 5'-phosphate synthase pdxT subunit	1.33
	pdx1	pyridoxal 5'-phosphate synthase pdxS subunit	1.51
	DHFS	dihydrofolate synthase	1.06
	phoA	alkaline phosphatase	1.02
	ilvE	branched-chain amino acid aminotransferase	1.09
	IDO, INDO	indoleamine 2,3-dioxygenase	1.43
	THI5	pyrimidine precursor biosynthesis enzyme	1.02
Glycolysis/Gluco neogenesis	*HXK2*	hexokinase isoenzyme	1.26
	PFK1	6-phosphofructokinase 1	1.29
	PYK2	pyruvate kinase	0.66
	ENO1	phosphopyruvate hydratase	1.62
	PDC1	pyruvate decarboxylase	0.81
	ALD4	mitochondrial aldehyde dehydrogenase	0.91
	ALD6	cytosolic aldehyde dehydrogenase	2.18
	ADH5	alcohol dehydrogenase isoenzyme	0.67

	ADH6	alcohol dehydrogenase	1.31
	ADH7	alcohol dehydrogenase	1.73
	LDH1	D-lactate dehydrogenase (cytochrome)	0.87
	CYB2	L-lactate dehydrogenase (cytochrome)	1.55
	ACS1	acetyl-CoA synthetase	2.55
	PYC1	pyruvate carboxylase	1.86
Ribosome	*RP-L8e*	large subunit ribosomal protein L8e	-1.87
	RP-S11e	small subunit ribosomal protein S11e	-1.67
	RP-L34e	large subunit ribosomal protein L34e	-1.47
	RP-S9e	small subunit ribosomal protein S9e	-2.20
	RP-SAe	small subunit ribosomal protein SAe	-1.60
Ribosome biogenesis in eukaryotes	*UTP22*	U3 small nucleolar RNA-associated protein 22	-1.74
	NOP1	rRNA 2'-O-methyltransferase fibrillarin	-1.66
	DCK1	H/ACA ribonucleoprotein complex subunit 4	-1.64
	IMP3	U3 small nucleolar ribonucleoprotein protein IMP3	-1.88
	POP3	ribonuclease P/MRP protein subunit POP3	-1.57
RNA polymerase	*RPC6*	DNA-directed RNA polymerase III subunit RPC6	-1.25
	RPA12	DNA-directed RNA polymerase I subunit RPA12	-1.55
	RPABC1	DNA-directed RNA polymerases I, II, and III subunit RPABC1	-1.31
	RPABC2	DNA-directed RNA polymerases I, II, and III subunit RPABC2	-1.27
	RPA34	DNA-directed RNA polymerase I subunit RPA34	-2.18
